# Influence of Role Models and Hospital Design on the Hand Hygiene of Health-Care Workers

**DOI:** 10.3201/eid0902.020249

**Published:** 2003-02

**Authors:** Mary G. Lankford, Teresa R. Zembower, William E. Trick, Donna M. Hacek, Gary A. Noskin, Lance R. Peterson

**Affiliations:** * Northwestern Prevention Epicenter, Chicago, Illinois, USA; † Northwestern Memorial Hospital, Chicago, Illinois, USA; ‡ Northwestern University Medical School, Chicago, Illinois, USA; § Centers for Disease Control and Prevention, Atlanta, Georgia, USA

**Keywords:** Handwashing, hand hygiene, sink access, new construction, role model, infection control, health-care worker behavior, research

## Abstract

We assessed the effect of medical staff role models and the number of health-care worker sinks on hand-hygiene compliance before and after construction of a new hospital designed for increased access to handwashing sinks. We observed health-care worker hand hygiene in four nursing units that provided similar patient care in both the old and new hospitals: medical and surgical intensive care, hematology/oncology, and solid organ transplant units. Of 721 hand-hygiene opportunities, 304 (42%) were observed in the old hospital and 417 (58%) in the new hospital. Hand-hygiene compliance was significantly better in the old hospital (161/304; 53%) compared to the new hospital (97/417; 23.3%) (p<0.001). Health-care workers in a room with a senior (e.g., higher ranking) medical staff person or peer who did not wash hands were significantly less likely to wash their own hands (odds ratio 0.2; confidence interval 0.1 to 0.5); p<0.001). Our results suggest that health-care worker hand-hygiene compliance is influenced significantly by the behavior of other health-care workers. An increased number of hand-washing sinks, as a sole measure, did not increase hand-hygiene compliance.

One of the key components for limiting spread of health-care–associated infectious disease is adequate infection control practice. A cornerstone of infection control is ensuring that health-care workers wash their hands at appropriate times. The Association for Professionals in Infection Control and Epidemiology (APIC), the Guidelines for Handwashing and Hospital Environmental Control (1985, 2001) from the Centers for Disease Control and Prevention (CDC), and the Hospital Infection Control Practices Advisory Committee each highlight specific indications for handwashing compliance (*1*–*4*). Although CDC guidelines state that handwashing is the single most important procedure to prevent nosocomial infection (*2*,*4*), studies continue to report unacceptable health-care worker hand-hygiene compliance rates (*5*–*12*). Efforts to improve hand-hygiene behavior that have focused on broad-based educational and motivational programs have had minimal sustained success (*11*–*14*).

Factors perceived as contributing to poor hand-hygiene compliance include unavailability of handwashing sinks, time required to perform hand hygiene, patient’s condition, effect of hand-hygiene products on the skin, and inadequate knowledge of the guidelines (*10*,*15*–*21*). In addition, some reports suggest that role models, group behavior, and the level of managerial support influence reported levels of compliance (*17*,*21*–*24*). One measure recommended to improve the hand-hygiene rate is enhanced access to hand-hygiene facilities (*15*–*17*,*25*). However, few studies have prospectively evaluated the association between hand-hygiene compliance and building design (*16*,*26*). We assessed the effect of medical staff role models and the number of health-care worker sinks on hand-hygiene compliance before and after construction of a new hospital designed for increased access to handwashing sinks. We also evaluated whether the frequency of health-care worker hand hygiene was influenced by the behavior of senior medical-care providers.

## Methods

### Setting and Study Participants

The old hospital had 683 private and semi-private rooms. Observations were made in the 33-bed hematology/oncology unit, the 23-bed solid organ transplant unit, the 16-bed surgical intensive-care unit (SICU), and the 11-bed medical intensive-care unit (MICU). Sink-to-bed ratios in the units were 8:33 in the hematology/oncology unit, 4:23 in the solid organ transplant unit, and 1:1 in both ICUs.

Sinks were located in various sites in the old hospital. The non-ICUs had a limited number of handwashing sinks for health-care worker use located on walls in the middle of each hallway, in clean storage rooms, and in soiled-linens utility rooms. The hematology/oncology unit had a single handwashing sink located in each of three hallways, two handwashing sinks located in each corridor for the bone marrow transplant patient rooms, and a handwashing sink in the anteroom to the bone marrow transplant suite. The solid organ transplant unit had a single handwashing sink located in each of two hallways. ICUs had private rooms with a sink located inside the entrance of every patient room but no hallway sinks.

The new hospital opened with 492 individual (private) patient rooms. Observations in the new facility were done in the 30-bed hematology unit, the 30-bed oncology unit, 30-bed solid organ transplant unit, the 12-bed SICU, and the 17-bed MICU. A sink dedicated for hospital personnel use is located inside every patient room. No sinks are available in the hallways.

### Hand-Hygiene Definition

We defined hand hygiene as any duration of washing with soap and water. No waterless alternatives were available for other types of hand hygiene during the study. We recorded hand-hygiene compliance on room entry and after each hand-hygiene opportunity. The definitions of hand-hygiene opportunities, patient contact, and invasive procedures used for this analysis are consistent with APIC or CDC guidelines (Table 1). Inanimate objects considered likely to be contaminated included endotracheal tubes, suction equipment, urinary collection devices, rectal tubes, thermometers, bed linens, and biohazardous waste containers.

**Table 1:** Definitions used to determine hand-hygiene opportunities, patient contact, and invasive procedures^a^

Hand-hygiene opportunities	Patient contact	Invasive procedures
Patient contact	Contact with patient’s skin	Phlebotomy
Performance of an invasive procedure	Contact with blood or body fluids	Intravenous or intramuscular injection of a medication
Placement of an intravascular device or urinary catheter	Contact with mucous membranes	Wound care
Visible soiling of hands		Urinary catheterization
Contact with body fluids		
Glove removal		
Contact with a likely contaminated environmental surface		

^a^Patient contact and invasive procedure are not mutually exclusive categories.

### Periods of Observation

During the two study periods, 1-hour observation periods were conducted weekdays between 8:00 a.m. and 5:00 p.m. The first observation period (period I; 25 weeks) took place from October 8, 1998, to April 29, 1999, in the old facility. The second observation period (period II; 24 weeks) took place in the new building from July 7 to December 23, 1999. A physician, two infection-control professionals, and a microbiologist were trained to individually observe, as follows: 1) protocol guidelines and study definitions were explained in detail from a printed handout that could be carried to the floor for reference during the study, and the data collection form was discussed; 2) the new observer accompanied the physician to a study unit and observed how to perform surveillance and complete the data collection form; 3) after observing several handwashing opportunities, the new observer made and recorded observations along with the physician; 4) the physician and new observer compared observations, and discrepancies were discussed to assess understanding of the protocol guidelines and study definitions; and 5) side-by-side comparisons were performed on a subsequent day, and the training was considered complete.

After entering the nursing unit, observers followed the initial worker or group of health-care workers they encountered who went into a patient room. To maintain health-care worker anonymity, individual identities were not recorded; therefore, we could not eliminate or control for repeat observations during analysis. The first persons entering a room were observed until departure from the room. We then went back into the hallway, and the next new persons entering a room were followed for the next observation, thus avoiding repeat observations of an individual health-care worker on any single observation day. Although health-care workers were not informed regarding the purpose of this study, if questioned during hand-hygiene observations, the investigators replied that infection-control measures were being monitored. No immediate feedback was provided to the health-care workers regarding hand-hygiene behavior.

Data gathered during observations included time of day, type and number of health-care workers entering the room, patient or equipment contact, compliance with hand-hygiene practices, glove use, invasive procedures, nursing unit and hospital, and whether isolation precautions had been posted. Health-care workers were designated as one of the following categories: physician; registered nurse; patient-care technician; respiratory, physical, or occupational therapist; pharmacist; radiology, electrocardiogram, or ultrasound technician; dietician; food service worker; unit secretarial staff; housekeeping staff; transportation staff; student; chaplain; volunteer staff; or technical sales support. Each physician was further classified as an attending or fellow, resident, or medical student.

A hierarchy was defined to assess the effect of other medical staff on hand-hygiene compliance. Health-care workers were ranked in the following order: 1) attending physician or fellow, 2) resident or intern, 3) nurse, 4) technical staff consisting of respiratory therapists, physical, or occupational therapists, radiology, electrocardiogram, ultrasound technicians, pharmacists, or dieticians, 5) patient-care technicians, and 6) housekeeping or transportation staff. Because the number of observations of medical students was small and determining the proper rank for medical students was difficult, we did not include students in the analysis of the influence of other personnel in the room. During analysis of the influence of other health-care workers present in the room, each health-care worker room entry was placed in one of the following categories: single person, highest ranking person in the room, in the room with a higher ranking person who performed hand hygiene, in the room with a higher ranking person who did not perform hand hygiene, in the room with a peer who performed hand hygiene, or in the room with a peer who did not perform hand hygiene. Peer status was defined as two or more health-care workers in the same category listed above (e.g., two nurses in the room together would be classified as peers). Hierarchy status was related to a purely theoretical belief of clinical expertise and knowledge held by the health-care worker.

Changes in accessibility and availability of hand-hygiene products and supplies between the two study periods included product modifications of soap, hand lotion, towels, and gloves. In general, products were more accessible in the new hospital. A para-chloro-meta-xylenol soap, (Medi-Scrub, Huntington Laboratories/Ecolab, St. Paul, MN) was the hand-hygiene agent in the ICUs and hematology/oncology unit in the old hospital. A 5-chloro-2-[2,4-dichlorophenoxyl, triclosan product (Healthstat, Richmond Laboratories, Huntington, IN) was used in the solid organ transplant unit in the old hospital and is now used in all units of the new hospital. An aloe vera lotion with triclosan was available in clean utility rooms of the old hospital (Accent Plus 1, Huntington Laboratories/Ecolab). In the new hospital, an amino lotion product is mounted at every health-care worker sink next to the soap dispenser (Tender Touch, Richmond Laboratories). Single-unit paper towel dispensers were available at each handwashing sink in both hospitals (Big Fold, Fort James Corp., Deerfield, IL, and Kleenex, Kimberly-Clark, Irving, TX, respectively). In the old hospital, various glove sizes were kept in clean utility rooms. In the new hospital, three sizes of gloves are located in wall-mounted dispensers next to towels and sinks. Powder-free gloves were available only for staff requiring them in the old hospital. Powder-free gloves are used exclusively in the new hospital by all personnel.

### Statistical Analysis

Data were collected on standardized forms and entered in Microsoft Excel (Microsoft Corp., Redmond, WA). To evaluate predictors for hand-hygiene compliance after a hand-hygiene opportunity, we compared categorical variables using chi-square or Fisher’s exact test; odds ratios (OR) and 95% confidence intervals (CI) were calculated by using Epi Info version 6.04c (*27*). All variables with p<0.1 by univariate analysis were evaluated by stepwise logistic regression for inclusion in the final logistic regression model. To evaluate the effect of group behavior on individual health-care workers, we performed a separate analysis. Using stepwise logistic regression, we constructed a model and entered all health-care worker groups into the final model. Single-person room entry was the referent group to which all other groups were compared. SAS software was used for all multivariate analyses (SAS Institute, Inc., Cary, NC).

## Results

### Observation Data

Observations were performed on 49 separate occasions for a total of 45 hours (range 9.6–13.5 h/U). A total of 560 health-care worker–patient interactions were observed, resulting in 729 hand-hygiene opportunities. A total of 305 (41.8%) hand-hygiene opportunities were observed in the old hospital and 424 (58.2%) in the new hospital. Of the 560 health-care worker–patient interactions observed, 237 (42.3%) of the workers were registered nurses, 190 (33.9%) were physicians, and 133 (23.8%) were other health-care workers. The old and new hospitals were similar in performance of invasive procedures and health-care worker type. In the older facility, health-care workers were more likely to wear gloves or touch a patient during the hand-hygiene opportunity.

Hand-hygiene compliance on room entry was significantly greater in the old hospital at 12% (36/304) compared to the new hospital at 6% (26/424) (p=0.006). After all hand-hygiene opportunities were assessed, we found that hand-hygiene compliance was significantly better in the old hospital compared to the new hospital (161/304 [53%] vs. 97/417 [23%]; p<0.001). Hand-hygiene compliance was significantly better after a hand-hygiene opportunity (258/721; 35.7%) compared to before a hand-hygiene opportunity (62/727; 8.5%; p<0.001). By univariate analysis, characteristics significantly associated with hand-hygiene compliance after a hand-hygiene opportunity included working at the old hospital, having patient contact, performing an invasive procedure, using gloves, and performing hand hygiene on room entry. A key finding was that when a higher ranking person in the room did not perform hand hygiene, other health-care workers were significantly less likely to wash their hands (Table 2).

**Table 2:** Comparison of characteristics for health-care workers who performed hand hygiene to those who did not perform hand hygiene, Northwestern Memorial Hospital

Variable	Hand hygiene		Odds ratio (95% confidence interval)	p value
	Yes (n=258) (%)	No (n=463) (%)		
Glove use	176 (68)	127 (27)	5.7 (4.0 to 8.0)	<0.001
Hand hygiene on room entry^a^	42 (16)	18 (3.9)	4.8 (2.6 to 8.9)	<0.001
Invasive procedure performed	34 (13)	25 (5.4)	4.4 (2.3 to 8.7)	<0.001
Old hospital	161 (62)	143 (31)	3.7 (2.7 to 5.2)	<0.001
Patient contact	130 (50)	132 (29)	2.6 (1.8 to 3.5)	<0.001
Nurse	135 (52)	219 (47)	1.2 (0.9 to 1.7)	0.2
Physician	60 (23)	127 (27)	0.8 (0.6 to 1.2)	0.2
In room with a higher ranking person who did not perform hand hygiene	12 (4.7)	77 (17)	0.2 (0.1 to 0.5)	<0.001

^a^Not recorded for a single observation.

During multivariate analysis, we identified the following independent predictors of hand-hygiene compliance: using gloves, performing an invasive procedure, working at the old hospital, performing hand hygiene on room entry, and having patient contact. Again, health-care workers present in the room with a higher ranking person or peer who did not perform hand hygiene were significantly less likely to wash their hands (Table 3).

**Table 3:** Comparison of characteristics and their effect on hand-hygiene compliance, by multivariate analysis^a^

Variable	Odds ratio (95% confidence interval)	p value
Glove use	3.5 (2.4 to 5.1)	0.003
Invasive procedure performed	2.7 (1.4 to 5.1)	0.003
Hand hygiene performed on room entry	2.4 (1.2 to 4.5)	0.01
Patient contact	2.1 (1.4 to 3.1)	<0.001
Health-care workers with a higher ranking health-care worker or peer who did not wash hands	0.4 (0.2 to 0.6)	<0.001
Hospital units^b^		
Old hospital, non-ICU	1.0	--
Old hospital, ICU	1.0 (0.6 to 1.8)	0.89
New hospital, non-ICU	0.4 (0.2 to 0.7)	0.002
New hospital, ICU	0.4 (0.2 to 0.7)	<0.001

^a^Hospital units grouped as intensive-care unit (ICU) or non-ICU units and by old or new hospital. All variables displayed in the table were included in the final model.

^b^All hospital unit groups were compared to the two non-ICUs in the old hospital, i.e., the referent group, which had the lowest sink-to-bed ratios (1:6 and 1:11). All other units had a sink-to-bed ratio of 1:1.

When we further evaluated group behavior, we found that compared to single person room entry, health-care workers in a room with a higher ranking person who did not wash were significantly less likely to wash their own hands. In each of these episodes, the higher ranking person was a physician or nurse. Surprisingly, if either a higher ranking person or peer was in the room and performed hand hygiene, then the frequency of hand hygiene for others in the group was no better than that of a room which only one person entered (Table 4). This observation suggests that the effect of a role model is highly significant but most potent in negatively influencing hand-hygiene behavior.

**Table 4:** Effect of behavior of other health-care workers in the room on health-care workers’ hand-hygiene compliance, by multivariate analysis, Northwestern Memorial Hospital^a^

Variable^b^	Odds ratio (95% confidence interval)	p value
Room entry alone (n=291)	1.0	–
In a room when a peer performs hand hygiene (n=48)	1.1 (0.6 to 2.3)	0.7
In a room when a higher ranking person performs hand hygiene (n=64)	0.8 (0.4 to 1.3)	0.3
Highest ranking person in the room (n=144)	0.6 (0.4 to 1.0)	0.07
In a room when peer does not perform hand hygiene (n=41)	0.4 (0.2 to 1.0)	0.05
In a room when higher ranking person does not perform hand hygiene (n=111)	0.2 (0.1 to 0.5)	<0.001

^a^Adjusted for variables significantly associated with increased hand-hygiene compliance, i.e., health-care worker glove use, hand hygiene on room entry, invasive procedures, patient contact, and old versus new hospital.

^b^Nurses and physicians accounted for most observations for all categories.

## Discussion

Despite construction of a new hospital with an increased number of sinks, we found that hand-hygiene compliance in the new facility decreased substantially. We demonstrated that health-care workers were significantly less likely to wash their hands if they were in a room with a peer or higher ranking person who did not perform hand hygiene. Not unexpectedly, hand-hygiene compliance was better after patient contact, performing an invasive procedure, and removing gloves.

Health-care workers were much less likely to perform hand hygiene if a peer or a higher ranking person in the room did not perform hand hygiene. Compared to health-care workers who entered a room alone, group behavior did not seem to improve if the higher ranking person or peer did wash their hands. Although these findings suggest that hand-hygiene behaviors can be affected by role model or peer hand-hygiene compliance, learned behaviors or time constraints may negatively influence group compliance with hand-hygiene procedures.

As suggested by some studies, physician hand-hygiene compliance has an impact on peer and group behaviors (*25*,*28*). A recent evaluation of learned physician behaviors found that only 8.5% medical student candidates washed after patient contact (*28*). Since medical students may someday be influencing future hand-hygiene compliance behaviors of other health-care workers, the importance of hand hygiene should be incorporated into the medical school curriculum.

Our observations also suggest that health-care worker hand-hygiene compliance may improve when health-care providers perceive risk for their own health. In particular, hand hygiene before patient contact in our study was significantly worse than hand hygiene after patient contact. Whereas patients may be protected from acquisition of pathogenic organisms if health-care workers perform hand hygiene before patient contact, health-care workers may perceive a risk to themselves after patient contact; they respond by washing their hands. In addition, health-care workers were more likely to perform hand hygiene after an invasive procedure, which does not benefit the individual patient, but rather the health-care worker, who may be concerned about acquiring a pathogen present in body fluids. Finally, glove use could be a marker for hand-hygiene compliance if health-care workers are concerned about the personal risk from transmission of pathogens, and thus are more likely to wear gloves and cleanse their hands.

The hand-hygiene compliance we observed (finding that nearly 50% of our workers washed their hands after patient contact) was similar to the frequency of hand-hygiene compliance reported by other investigators (*5*,*7*,*10*–*12*). Even though we saw no improvement, our baseline rate was comparable to that of a recent report by Bischoff and associates after they improved hand hygiene compliance by using accessible alcohol-based antiseptics and increased hand-hygiene compliance (41% to 48%) after patient contact (*5*).

While some studies (*5*,*15*), and health-care worker surveys (*29*) suggest that sink access is an important determinant of hand-hygiene compliance, we found access is not the sole requirement needed to increase hand-hygiene compliance. Few reports address the impact of hospital design on hand-hygiene compliance. Kaplan and McGuckin (*15*) compared two units and demonstrated a greater hand-hygiene frequency among nurses in an MICU having a 1:1 sink-to-bed ratio compared with an SICU having a 4:1 sink-to-bed ratio (76% vs. 51%; p<0.01). However, a study by Preston and colleagues evaluating hand hygiene after the number of sinks in an ICU was increased found that improved sink access had no effect on hand-hygiene frequency (*26*). Possible explanations for the decreased hand-hygiene compliance we observed include: 1) more patient-days (5.2%) and more admissions (11.7%) per month occurred for study period II compared to study period I; 2) disrupted work flow because of the new and unfamiliar environment of the new hospital; 3) removal of hallway sinks; and 4) addition of new or temporary nursing staff because of the increased number of patients. Patient:nurse ratios are considered an important determinant in hand-hygiene compliance (*6*). In our study, we believe the ratios were similar in the old and new hospitals, but these data were only formally available for the ICUs. The average patient:nurse ratio during the observation periods in the new MICU was 1.42 (standard deviation [SD] 0.15) and in the old MICU, the ratio was 1.43 (SD = 0.05). We found similar data in the SICU areas, where average patient:nurse ratio during our observation periods in the new unit was 1.03 (SD = 0.24); in the old unit, the ratio was 1.22 (SD = 0.14). Thus, the ICU staffing patient:nurse ratio was similar during the observation shift for the ICUs in the old and new hospitals (p>0.2).

Because of changes in unit size throughout the new hospital, the study units used nursing staff from areas of the hospital with decreased staff requirements. Nursing personnel were reassigned to appropriately staff the newly configured larger patient-care areas. Specifically, nurses were transferred to units requiring the same education and skill sets (i.e., medical unit to medical unit, surgical unit to surgical unit, and intensive care to intensive care). To ensure adequacy of training, institutional education activities are systematic. All new employees are required to attend a hospital orientation that includes general information on infection-control issues. On the first day of work in the department, new and transferred employees also participate in individual departmental orientation. The orientation includes a review of policies and procedures, job-specific responsibilities, performance expectations, and unit-specific infection control measures (Northwestern Memorial Hospital Orientation Program, Human Resources Policy 4.96). For agency and float pool nursing staff, a 3-hour orientation is provided, which includes a review of policies, procedures, and paperwork. Float pool nurses have at least 1 year of experience before being hired and are required to complete annual competencies that incorporate infection-control measures. While they are used throughout the hospital, only nurses having cardiac-care certification may work in the ICUs.

Our study had several limitations. First, the two study periods were in different seasons and involved different house staff. A greater proportion of observations in the older hospital were conducted in the winter months, which we postulated would decrease hand-hygiene compliance because of increased skin dryness (*30*,*31*). However, hand-hygiene compliance was better in the old hospital, despite more observations’ being performed during the winter. Hand-hygiene rates were consistently lower in the new hospital for all types of health-care workers; a change in house staff (residents) was unlikely to have influenced our overall results. While nurse-to-patient ratios were not specifically measured on all units, we noted no obvious change in staffing levels, and when we compared ICUs, the staffing ratios were similar (p>0.2). Since we did not perform observations at night or on the weekends, and duration or efficacy of hand hygiene was not evaluated during our study, we cannot comment on hand-hygiene compliance for these shifts or on the effectiveness of health-care workers’ hand-hygiene technique.

Most units changed soap products between the old and new facilities, with the exception of the solid organ transplant unit. On this unit, the handwashing compliance dropped from 62% in the old hospital to 23% in the new hospital (data not shown); however, the decrease was similar to that observed in other monitored units, which suggests any change in soap product was not the major factor. Additionally, a change from powdered to nonpowdered gloves may have negatively influenced hand hygiene. Reviewing our data set for the potential influence of this factor, we found that if gloves were worn and removed, then hand-hygiene frequency in the old hospital was 131/176 (74%), whereas in the new hospital, the frequency was 76/128 (59%) (p=0.005). If gloves were not worn, then hand-hygiene frequency in the old hospital was 30/116 (26%), and in the new hospital 21/260 (8%) (p<0.001). Thus, both the absolute and relative decreases in hand-hygiene frequency were greater for the nongloved health-care workers, which suggests that powder in the gloves was not the reason for diminished hand hygiene.

Pittet has posed the problem of hand-hygiene compliance: How can we change the behavior of health-care workers and how can we maintain such a change (*32*)? We strongly agree and believe the time has come to “think outside the box” for solutions to poor hand hygiene by health-care workers. Obtaining simple feedback by measuring soap and paper towel levels was recently shown not to have an impact (*33*). Our observations also show that another straightforward measure—improving health-care worker access to sinks—used as a sole measure, does not result in increased hand-hygiene compliance. However, the new facility is now ideally equipped to determine what is needed to improve hand-hygiene performance among health-care workers (*34*). To substantially improve hand-hygiene compliance, additional factors must be considered, including improving health-care workers’ skin conditions and using alcohol-based alternatives (a factor recently demonstrated to improve hand-hygiene compliance [*35*]), focusing on educational interventions, and providing administrative support. Since hand-hygiene compliance was significantly worse in groups where a ranking member of the group did not perform hand hygiene, a greater focus on improving compliance among physicians and nurses who are important role models may also result in better hand-hygiene compliance among all health-care workers.
